# Persistent metallic Sn-doped In_2_O_3_ epitaxial ultrathin films with enhanced infrared transmittance

**DOI:** 10.1038/s41598-020-61772-y

**Published:** 2020-03-18

**Authors:** Dongha Kim, Shinbuhm Lee

**Affiliations:** Department of Emerging Materials Science, Daegu-Gyeongbuk Institute of Science and Technology, Daegu, 42988 Republic of Korea

**Keywords:** Materials for devices, Electronics, photonics and device physics

## Abstract

Infrared transparent electrodes (IR-TEs) have recently attracted much attention for industrial and military applications. The simplest method to obtain high IR transmittance is to reduce the electrode film thickness. However, for films several tens of nanometres thick, this approach unintentionally suppresses conduction due to surface electron scattering. Here, we demonstrate low sheet resistance (<400 Ω □^−1^ at room temperature) and high IR transmittance (>65% at the 2.5-*μ*m wavelength) in Sn-doped In_2_O_3_ (ITO) epitaxial films for the thickness range of 17−80 nm. A combination of X-ray spectroscopy and ellipsometry measurements reveals a persistent electronic bandstructure in the 8-nm-thick film compared to much thicker films. This indicates that the metallicity of the film is preserved, despite the ultrathin film configuration. The high carrier mobility in the ITO epitaxial films further confirms the film’s metallicity as a result of the improved crystallinity of the film and the resulting reduction in the scattering defect concentration. Thus, ITO shows great potential for IR-TE applications of transparent photovoltaic and optoelectronic devices.

## Introduction

With advances in optoelectronics, transparent electrodes (TEs) have made many novel functionalities possible^1–3^. Conventional TEs provide high transmittance for visible light; however, they are opaque at infrared (IR) wavelengths due to the free electron response. Recently, there has been renewed interest in high IR transparency for smart windows, solar cells, sensors, and military applications^4^. Various materials, such as carbon-based nanomaterials^5^, have been studied as potential IR-TE candidates; however, these materials do not provide the desired properties for optimal performance.

Light propagating through a material can be described by its optical intensity *I*(*z*) at position *z*, as given by Beer’s law, $I(z)={I}_{o}{e}^{-\alpha z}$, where *I*_*o*_ is the optical intensity at *z* = 0 and the absorption coefficient *α* quantifies the absorption of light by the material^6^. According to this law, the simplest method to obtain high IR transmittance involves thickness reduction. However, when the thickness of films is less than several tens of nanometres, conduction in the film is suppressed by electron scattering near the surface. Thus, there is a trade-off between high transparency and low conduction in thin films.

Sn-doped In_2_O_3_ (ITO) is a widely used TE, e.g., for the deposition of ferroelectric HfO_2_ and photovoltaic BiVO_4_ epitaxial films on Y-stabilized ZrO_2_ (YSZ) substrates^7,8^. We chose ITO in the current study, as it was recently revealed that In_2_O_3_-based materials offer a high carrier mobility *μ* > 20–100 cm^2^ V^−1^ s^−1^ (ref. ^1^). According to the free electron model, a high *μ* facilitates considerable conductivity *σ* (= *eNμ*, where *e* and *N* are the electrical charge and carrier density, respectively). Furthermore, by doping In_2_O_3_ with either a transition metal^9^ or hydrogen^10,11^, In_2_O_3_ can exhibit a higher *μ* (~130 cm^2^ V^−1^ s^−1^), with a moderate carrier density of ~10^20^ cm^−3^. Native defects in polycrystalline and amorphous films disturb the physical mechanism responsible for the intrinsic IR-TE properties of In_2_O_3_^12,13^. There have been few studies on high-quality In_2_O_3_ as epitaxial films. In addition, the measurement of the IR-TE performance in In_2_O_3_ has been limited to below 2.5 *μ*m. Here, we considered the wavelength range up to 10 *μ*m.

The purpose of this study was to examine the intrinsic IR-TE performance of single-crystalline ITO epitaxial films as the film thickness was varied. We found that ITO epitaxial films have high IR transmittance (> 65% at the 2.5-*μ*m wavelength) and low sheet resistance (<400 Ω □^−1^) at room temperature for the thickness range of 17−80 nm. X-ray photoemission/absorption spectroscopy (XPS/XAS) and spectroscopic ellipsometry (SE) measurements showed that persistent metallic transport was supported by the electronic bandstructure and the absence of extrinsic defects. We compared the IR-TE performance of single-crystalline ITO films with those of other TEs.

### Single-crystalline Sn-doped In_2_O_3_ (ITO) epitaxial films

**Figure 1:**
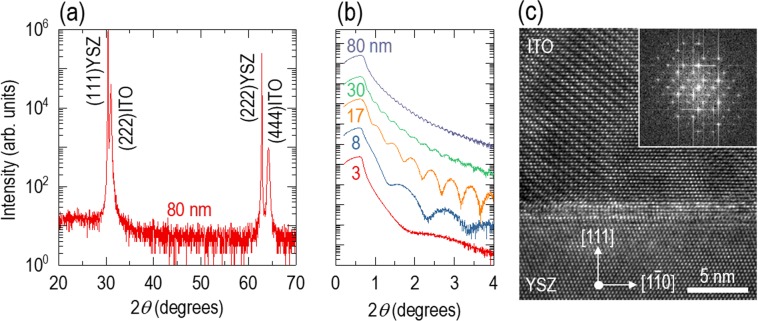
Single-crystalline Sn-doped In_2_O_3_ (ITO) epitaxial films grown on (111)-oriented Y-stabilized ZrO_2_ (YSZ). (**a**) X-ray diffraction *θ*−2*θ* scan of an 80-nm-thick ITO film. There are four peaks corresponding to {111}YSZ and {222}ITO, indicating epitaxial growth of the ITO film. (**b**) X-ray reflectivity of various thickness ITO epitaxial films. There are clear fringe patterns for all thicknesses, indicating that the interface and surface are smooth. (**c**) Cross-sectional transmission electron microscopy image of the 80-nm-thick film. It shows a well-ordered atomic arrangement and a smooth interface. The fast Fourier transform image also reveals single-crystalline growth of the cubic ITO epitaxial film.

The X-ray diffraction (XRD) *θ*−2*θ* scan of an 80-nm-thick ITO film shown in Fig. 1a features two peaks at 2*θ* = 31.1° and 64.2°, corresponding to diffraction from the (222) and (444) planes of ITO, respectively. With the exception of the peaks at 2*θ* = 30.4° and 62.8° generated by diffraction from the (111) and (222) planes of YSZ, we did not find any additional peaks, indicating the high quality of our ITO epitaxial films [Fig. S1 shows XRD *θ*−2*θ* scan results for other film thicknesses]. The crystal structure of ITO is cubic bixbyite (space group of Ia$\bar{3}$), with a lattice constant of *a*_ITO_ = 10.12 Å. On cubic fluorite YSZ (Fm$\bar{3}$m) with a lattice constant of *a*_YSZ_ = 5.13 Å, we could grow ITO epitaxial films due to the relatively small lattice mismatch ($=\frac{2\times {a}_{{\rm{YSZ}}}-{a}_{{\rm{ITO}}}}{2\times {a}_{{\rm{YSZ}}}}\times 100$) of +1.4%, in which the positive sign indicates that an in-plane tensile strain is applied to the ITO films. Therefore, the YSZ substrate provides an interface commensurate with ITO epitaxial films, minimizing defect formation^14^. Figure 1b shows a clear fringe patterns in the X-ray reflectivity for ITO epitaxial films with 3–80 nm thicknesses. This highlights that all the films used in this work have a commensurate interface and a flat film surface. Figure 1c shows a cross-sectional transmission electron microscopy (TEM) image of an 80-nm-thick ITO epitaxial film. The well-ordered atomic arrangement in the TEM image indicates few grain boundaries and dislocations in the region far from the ITO/YSZ interface. The fast Fourier transform images of ITO epitaxial films also revealed their single-crystalline growth with a cubic structure. On the other hand, within a few unit cells near the interface in the cross-sectional TEM image, we observed a few defects, formed to release even the small misfit strain between ITO and YSZ, as also proven by the XPS in Fig. S2. The cross-sectional TEM image taken over a wide area shows not only a well-ordered atomic arrangement in the ITO film but also a flat film surface (Fig. S3a). Atomic force microscopy of the 80-nm-thick ITO film revealed a root-mean-square surface roughness of 2.3 nm (Fig. S3b).

### Enhanced infrared transmittance of ITO with thickness reduction

**Figure 2:**
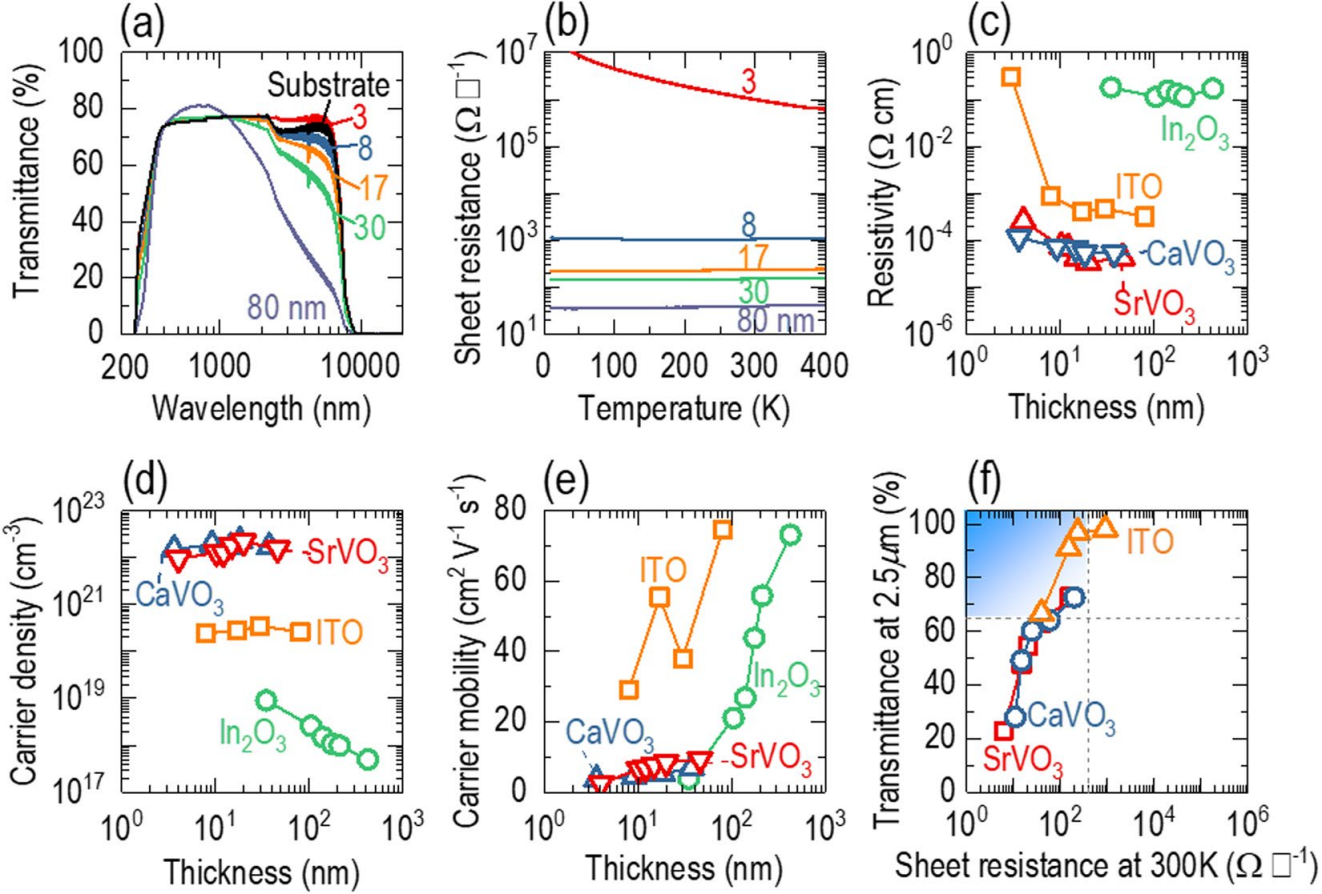
(**a**) Transmittance of ITO epitaxial films as a function of wavelength. With a decrease in the film thickness from 80 to 3 nm, the transmittance increases over a wide range of wavelengths (300–10,000 nm). At the 2.5-*μ*m wavelength, films thinner than 80 nm show high transmittance (> 65%). (**b**) Sheet resistance as a function of temperature. With a decrease in the film thickness, the sheet resistance increases. At room temperature, films thicker than 8 nm show low sheet resistance (<1,000 Ω □^−1^). (**c**) Resistivity as a function of thickness. The resistivity is almost the same over the film thickness range of 8–80 nm but abruptly increases in the 3-nm-thick film. The resistivity of ITO is much lower than that of In_2_O_3_ and much higher than those of CaVO_3_ and SrVO_3_. (**d**) Carrier density as a function of thickness; ITO epitaxial films have an carrier density of 10^20^~10^21^ cm^−3^. (**e**) Carrier mobility as a function of thickness. The 80-nm-thick film shows a very high mobility of ~75 cm^2^ V^−1^ s^−1^, which abruptly decreases with decreasing film thickness. Nevertheless, the mobility of ITO is much higher than those of CaVO_3_, SrVO_3_, and In_2_O_3_ for all thickness ranges. (**f**) Optimal thickness of ITO epitaxial films for IR-TEs. We plotted the transmittance versus the sheet resistance. The region highlighted in blue indicates the films that satisfy both the transmittance and sheet resistance requirements (>65% and <400 Ω □^−1^, respectively). ITO epitaxial films are in this region for 17−80 nm thicknesses; CaVO_3_ and SrVO_3_ are in this region for films thinner than 10 nm. Data for CaVO_3_, SrVO_3_, and In_2_O_3_ are taken from refs. ^18,19^, respectively.

With decreasing film thickness, the ITO epitaxial films became transparent in the IR range for wavelengths up to 10 *μ*m. Figure 2a shows the transmittance for various thicknesses as a function of wavelength (200–20,000 nm). We directly measured the transmittance by penetrating light through ITO films grown on double-side polished YSZ substrates. The 80-nm-thick film showed high transmittance in the visible range (400–700 nm), as typically observed in bulk ITO. The transmittance was abruptly suppressed in the ultraviolet (<400 nm) range due to interband transitions and in the IR range (>1,000 nm) due to the free electron response. When we reduced the film thickness, the transmittance showed a gradual increase over the entire wavelength range, consistent with Beer’s law. It should also be noted that the transmittance of the ITO films was occasionally greater than that of the YSZ substrate, e.g., the transmittance for 400−1,000-nm wavelengths. This unusual behaviour probably occurred due to the antireflection effect^15^. The transmittance of the 3-nm-thick film was limited by that of the YSZ substrate. For films thinner than 30 nm, the transmittance exceeded 65% at the 2.5-*μ*m wavelength, showing strong potential for IR-TE applications.

### Persistent metallicity in ultrathin ITO films

Although the transmittance is enhanced in ultrathin films, the conductivity is empirically suppressed due to electron scattering near the film surface. To explore the fundamental thickness limit for the metallic properties of our various thickness ITO epitaxial films, we measured the sheet resistance as a function of temperature (Fig. 2b). The sheet resistance of the 80-nm-thick film increased with temperature, indicating a typical metallic nature over the entire temperature range. The sheet resistances of the 8-, 17-, and 30-nm-thick films showed a metal-to-insulator transition upon cooling (Fig. S4), which has usually been attributed to the appearance of weakly localized states, i.e., interfacial defects in this work. In addition, it is quite natural that the sheet resistance of the ITO epitaxial films increased with decreasing film thickness. The sheet resistance of the 8-nm-thick ITO film became two orders of magnitude larger than that of the 80-nm-thick film. However, the resistivity was still less than 10^−3^ Ω cm (Fig. 2c), which is close to the Mott–Ioffe–Regel limit^16^, indicating that the 8-nm-thick film is on the verge of attaining a metallic nature near room temperature. The 3-nm-thick film lost metallicity, based on the reduction in the sheet resistance with increasing temperature, over the full temperature range of our experiment. The abrupt increase in the resistivity for the 3-nm-thick film provides a lower limit to the film thickness for IR-TE applications that could be considered for our ITO films. Additionally, the mean free path (~8 nm) of our ITO epitaxial films was much shorter than that of conventional metals, such as Au (~50 nm) and Ag (~52 nm)^17^, but longer than that of correlated transparent conductors, such as CaVO_3_ (~3.9 nm) and SrVO_3_ (~5.6 nm)^18^.

To understand the persistent metallicity of the ITO ultrathin epitaxial films down to an 8-nm thickness, we measured the carrier density and mobility, key metrics of transparent conductors. Figure 2d shows that our ITO epitaxial films have an carrier density of 10^20^−10^21^ cm^−3^. This value is much higher than that of In_2_O_3_ (10^18^–10^19^ cm^−3^)^19^, as Sn doping provides one electron in part, and is lower than that of SrVO_3_ and CaVO_3_ (10^22^ cm^−3^)^18^. As shown in Fig. 2e, our ITO epitaxial films showed higher mobilities (e.g., ~75 cm^2^ V^−1^ s^−1^ for an 80-nm film thickness) than those of In_2_O_3_, SrVO_3_, and CaVO_3_. The typical mobility values of amorphous ITO thick films encountered in the literature lie at approximately 10 cm^2^ V^−1^ s^−1^ (ref. ^1^); thus, we inferred that the high mobility in our film is due to the improved crystallinity induced by epitaxial growth. High density and high mobility, which may be attributed to improved crystallinity, promote metallicity even in ultrathin ITO epitaxial films. While the density does not significantly vary with a reduction in the film thickness, the mobility decreases with decreasing thickness. Nevertheless, the mobility is still higher than those of In_2_O_3_ and vanadates. As electron scattering by interfacial vacancies is amplified when the film is thinner, the mobility may be reduced with a reduction in the film thickness.

### Potential of ITO films for infrared transparent electrodes

Persistent metallicity, as well as high IR transparency, enables the use of single-crystalline ITO films as IR-TEs. The transmittance and sheet resistance are characteristic parameters of TE performance. To determine the optimal thickness range of ITO for IR-TE applications, we plotted the transmittance as a function of sheet resistance, as shown in Fig. 2f. To avoid the contribution of the substrate to the transmittance of ITO, we extracted the transmittance *T* (=*I*_*t*_/*I*_0_) by normalizing the intensity *I*_*t*_ transmitted through the ITO film and the YSZ substrate to the *I*_0_ of the bare YSZ substrate^20^. We examined the feasibility of IR-TEs on the basis of *T* > 65% at a wavelength of 2.5 *μ*m and sheet resistance *R*_*s*_ < 400 Ω □^−1^ at room temperature, as highlighted in the graded blue colour [although we investigated the transmittance up to the wavelength of 10 *μ*m, we compared the data at 2.5 *μ*m since most TE materials showed transmittance below 2.5 *μ*m]. The 17−80-nm-thick ITO epitaxial films satisfied both requirements, and more precisely, the lower limit of the film thickness was between 8 and 17 nm in our experiment. The transmittance above this thickness range and the sheet resistance below this thickness range were limiting factors for IR-TE applications. It should be noted that IR-TEs based on SrVO_3_ and CaVO_3_ were available only for films thinner than 10 nm. Above this thickness, the transmittance is limited, while the sheet resistance is very low [see Fig. S5 for the thickness dependence of the sheet resistance and transmittance of ITO, SrVO_3_, and CaVO_3_]. Thus, our ITO films within the thickness range of 17 < *t* < 80 nm, with *T* > 65% at the 2.5-*μ*m wavelength and *R*_*s*_ < 400 Ω □^−1^ at room temperature, are encouraging for IR-TE applications.

### Electronic bandstructures of ITO epitaxial films

The electronic bandstructure has been widely used to describe the unique interplay between the conductivity and transmittance in TEs. The valence band of ITO is typically composed of occupied O-2*p* antibonding states, and the conduction band is primarily composed of unoccupied In-5*s* bonding states^2^. Strong hybridization between the O-2*p* and In-5*s* orbitals induces high carrier mobility due to the low effective mass and low optical absorption resulting from the large bandgap and low density of states in the conduction band. Sn dopants can donate one excess electron, enhancing the conductivity of In_2_O_3_. To resolve the valence band, conduction band, and interband transitions in the various thickness ITO epitaxial films, we carried out XPS, XAS, and SE measurements at room temperature.

**Figure 3:**
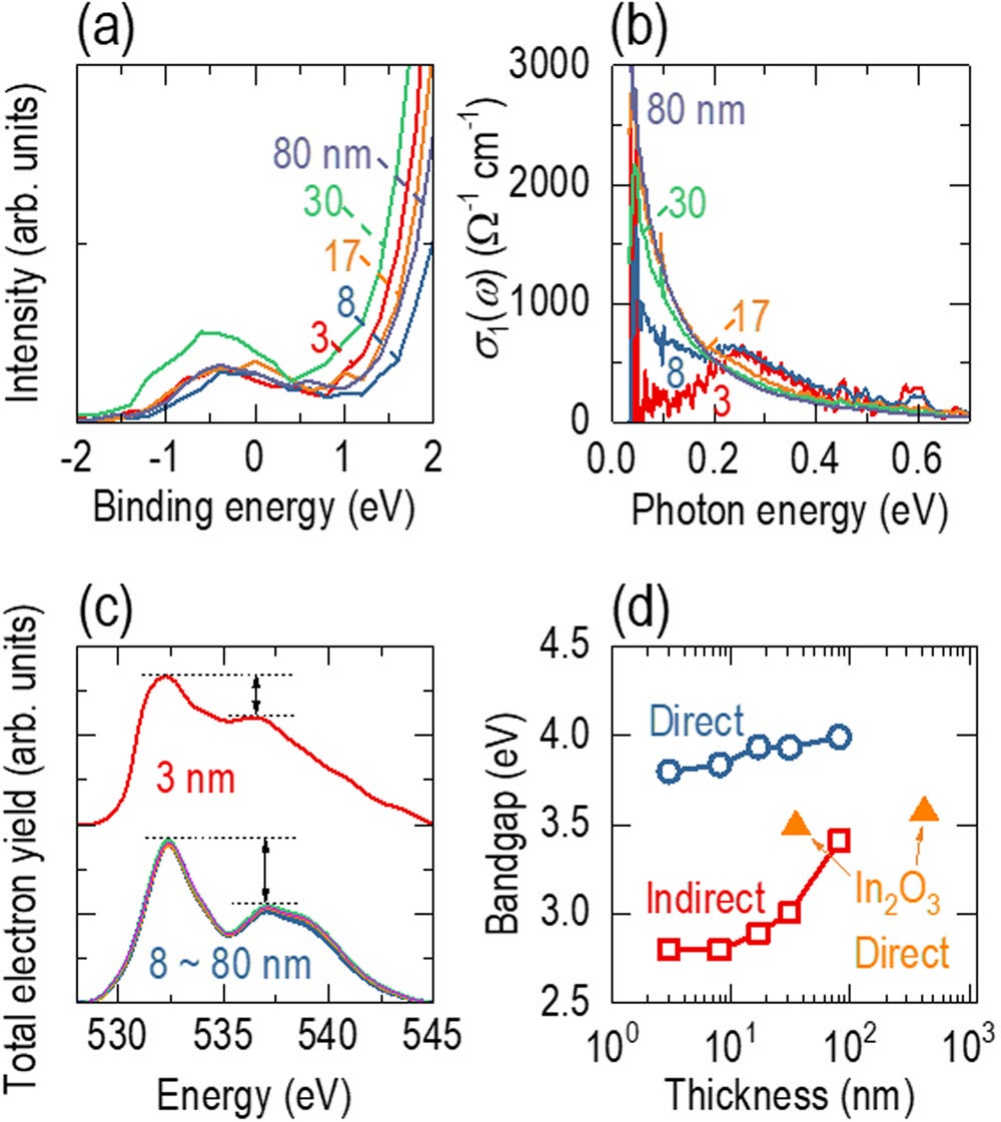
Electronic bandstructures of ITO epitaxial films with various film thicknesses. (**a**) XPS intensity near the Fermi level. We observed a strong signal near the Fermi level for all films. (**b**) Optical conductivity as a function of photon energy. There is a nonnegligible spectral weight near a very small photon energy (~0.03 eV), even in the 3-nm-thick film. (**c**) X-ray absorption spectroscopy (XAS) of the total electron yield to investigate the conduction band. The XAS spectra do not significantly change as the film thickness approaches 8 nm, indicating preservation of the conduction band. We highlight the change in the absorption ratio by the length of the arrows. (**d**) Bandgap as a function of film thickness. The indirect and direct bandgaps decrease with decreasing film thickness. The direct bandgap of In_2_O_3_ epitaxial films taken from ref. ^19^ shows a similar trend.

We observed strong XPS intensity near the Fermi level, independent of the film thickness (Fig. 3a), indicating that metallic regions may exist, even in the 3-nm-thick film. We also found a similar metallic character when we measured the optical conductivity of the 3-nm-thick film (Fig. 3b). Metals typically show strong spectral weight at low photon energy (<1 eV) due to the free electron response. Thick films (>17 nm) showed strong absorption near zero photon energy; however, the absorption was suppressed for the 8-nm-thick film. Surprisingly, the 3-nm-thick film still had a nonnegligible spectral weight near a very small photon energy (~0.03 eV), indicating a persistent metallic nature in ultrathin films. The discrepancy between the insulating transport (Fig. 2a) and metallic characters in the XPS and ellipsometry results for the 3-nm-thick film indicates that the metallic states may adopt a ‘puddle’ configuration^21^, as defects in proximity to the ITO/YSZ interface might disconnect the metallic regions in ultrathin films.

The conduction band does not show any significant changes with a reduction in the film thickness down to 8 nm. Figure 3c shows the XAS total electron yield of the various thickness ITO films. Hybridized O-2*p* and In-5*s* orbitals are positioned in the photon energy range from 528 to 545 eV, which corresponds to X-ray absorption from core levels into unoccupied levels in the conduction band. The overall spectra are similar to other results reported in the literature^22,23^. We found that the peaks were preserved in the 8–80-nm-thick films. This observation is consistent with density functional theory (DFT) calculations for In_2_O_3_ epitaxial films, indicating that the conduction band does not significantly change with variation in the film thickness^19^. However, the 3-nm-thick film showed a smaller absorption ratio (indicated by arrows) than the thicker films, also supporting the existence of defects at the ITO/YSZ interface.

We measured the thickness-dependent bandgap by plotting the absorption coefficient as a function of photon energy [see Fig. S6 for the bandgap calculation]. As shown in Fig. 3d, the indirect bandgap for the thicker films, e.g., 3.4 eV for 80 nm, was in general agreement with other reported values of ~3.5 eV^19^. The direct bandgap of the ITO epitaxial films, 4.0 eV for 80 nm, was higher by ~0.5 eV than that of In_2_O_3_ epitaxial films^19^. As the film thickness was reduced, the indirect and direct bandgaps decreased to 2.8 and 3.8 eV, respectively. The reduction in the direct bandgap was very small (<0.1 eV) with decreasing film thickness from 80 to 17 nm, and most of the reduction occurred over the reduction range of 17 to 3 nm. This two-step shift arises from different sources. The small reduction observed as the film thickness decreased from 80 to 17 nm is consistent with the observation (0.08 eV) in thick In_2_O_3_ epitaxial films when the thickness decreased from 420 to 35 nm (triangles in Fig. 3d); DFT calculations also indicated a small change of 0.06 eV^19^. The significant decrease in the bandgap of thinner films is most likely due to defects near the interface.

## Conclusion

Our results showed that ITO epitaxial films are IR transparent (*T* > 65% at the 2.5-*μ*m wavelength) and conducting (*R*_*s*_ < 400 Ω □^−1^) at room temperature in the thickness range of 17 < *t* < 80 nm. This thickness range is broad compared with those for SrVO_3_ and CaVO_3_, which are IR transparent below 10 nm. With a reduction in the film thickness, we observed enhanced transmittance, as expected from Beer’s law; however, the conductivity was limited. The excellent IR-TE performance of our ITO films is attributable to the high carrier mobility in the films, in which improved crystallinity is the key factor, as electrons are easily scattered by defects. The high-crystallinity, epitaxial, ultrathin ITO films retained sufficient conductivity. The preservation of the electronic bandstructure from the bulk to a film thickness of 8 nm supports a persistent metallicity in the ITO ultrathin films. The YSZ buffer layer was used to grow epitaxial films on (001)-oriented silicon, e.g., the VO_2_(M1) phase showing the metal–insulator transition^24^ and ferroelectric HfO_2_ (ref. ^25^). Our work provides insights into the epitaxial growth of ITO on silicon substrates with a YSZ buffer layer. Thus, IR transparent and conducting ITO films show great potential for IR-TE applications and transparent photovoltaic and optoelectronic devices.

## Methods

### Deposition of ITO epitaxial films on (111)YSZ

To reduce the defects and phase separation that induce impurity scattering, we fabricated single-crystalline ITO films on (111)-oriented YSZ using pulsed laser epitaxy. We made a polycrystalline ITO target by sintering pelletized In_2_O_3_:SnO_2_ powder (90:10 wt.%, Thermo Fisher Scientific) for 12 hours at 1,200 °C. We evaporated the ITO target towards (111)-oriented 9.5 mol% Y_2_O_3_-stabilized ZrO_2_ substrates (CrysTec GmbH) by pulsed laser epitaxy. For target evaporation, we repeatedly pulsed the laser beam on the target with an intensity of 1 J cm^−2^ at a rate of 5 Hz using a KrF excimer laser with a 248-nm wavelength (model IPEX-760; LightMachinery Inc.). During film deposition, the substrate was heated to 600 °C in an oxygen gas atmosphere at a pressure of 10 mTorr. By fitting the XRR fringe patterns with the X’Pert reflectivity program, we obtained the film thickness. The deposition rate of the ITO film was 0.16 Å per pulse. The fringe pattern of the 3-nm-thick film was too broad (Fig. 1b); thus, the film thickness was calculated by multiplying the laser pulse count by the growth rate of 0.16 Å per pulse. We investigated the structural quality by performing XRD *θ*−2*θ* scans in a four-circle high-resolution XRD instrument (model Empyrean; PANalytical) using Cu radiation with a wavelength of 1.54 Å. We used the same equipment for XRR to estimate the surface roughness and film thickness and for reciprocal space mapping to evaluate the strain level of ITO epitaxial films on YSZ substrates. We obtained cross-sectional images by TEM (model Tecnai G^2^ 20; FEI). To investigate the surface topology, we used an atomic force microscope (model XE7; Park systems). The scan area and rate were 10×10 *μ*m^2^ and 0.5 Hz, respectively.

### Measurement of transmittance

To directly measure the transmittance, we employed two spectrometers in the wavelength range of 175–3,300 nm (Cary 5000 UV-Vis-NIR, Agilent Technologies) and 1,282–28,571 nm (Nicolet Continumm, Thermo Scientific). We used an empty hole to define the reference transmittance of ITO films grown on double-side polished YSZ substrates.

### Measurement of electrical properties

We used a physical property measurement system (Quantum Design Inc.) to investigate the direct current transport properties over the temperature range of 10–400 K. We calculated the sheet resistance by dividing the measured resistivity by the film thickness (ref. ^26^). To calculate the carrier density and mobility, we measured the Hall coefficient via the van der Pauw method. Applying a constant current, the Hall voltage was measured with respect to the magnetic field strength (−4 to 4 T).

### Measurements to establish the electronic structure

To establish the electronic structure, we performed the following measurement. We carried out XPS over the binding energy range of −5 to 10 eV to investigate the valence band near the Fermi level. The XPS instrument (model ESCALAB 250Xi; Thermo Scientific) was operated with a monochromatic Al source under an environmental pressure of 10^−8^ Torr. We used the C-1*s* peak at 284.5 eV as an energy reference. The optical conductivity *σ*_1_(*ω*) can be calculated by ${\sigma }_{1}(\omega )={{\epsilon }}_{0}\omega {e}_{2}$ (ref. ^6^), where *e*_2_ is the imaginary part of the complex relative dielectric constant. We used optical constants, normal refractive index *n* and extinction coefficient *κ* to calculate *e*_2_ ($=2n\kappa$)^6,18^. *n* and *κ* were determined using spectroscopic ellipsometry by calculating $\phi =\frac{2\pi nt}{\lambda }$ and $\psi ={\tan }^{-1}\frac{2\kappa }{{n}^{2}+{\kappa }^{2}-1}$, where *λ* represents the wavelength of the incident light. *n* and *k* were also used to determine the absorption coefficient $\alpha =\frac{4\pi \kappa }{\lambda }$. We employed ellipsometry in the photon energy range of 0.03–0.73 eV (IR-VASE; J.A. Woollam Co.) and 0.73–6.42 eV (M-2000DI; J. A. Woollam Co.). We used 0.002 eV and 0.05 eV step sizes for the former and the latter, respectively. After checking invariant spectra for an incident angle of 60°, 65°, or 70°, we used 70° for all ellipsometry measurements. We performed XAS in total electron yield mode at the 2A Beamline of the Pohang Accelerator Laboratory.

## Supplementary information

Supplementary information.
