# Supplementary material for: Persistent metallic Sn-doped In2O3 epitaxial ultrathin films with enhanced infrared transmittance
Source: Sci Rep. 2020 Mar 18;10:4957. doi: 10.1038/s41598-020-61772-y (PMC7080801; doi:10.1038/s41598-020-61772-y)
Supplement: Supplementary file 1 — Supplementary information. [file 41598_2020_61772_MOESM1_ESM.pdf]

Supplementary information

Persistent metallic Sn-doped In_2O_3 epitaxial films with enhanced infrared transmittance

*Dongha Kim and Shinbuhm Lee**

Department of Emerging Materials Science, Daegu-Gyeongbuk Institute of Science and Technology, Daegu 42988, Republic of Korea.

*E-mail: lee.shinbuhm@dgist.ac.kr

1. X-ray reflectivity of 3–80-nm-thick films

Figure S1 shows X-ray diffraction θ–2θ scans for Sn-doped In_2O_3 (ITO) films with thicknesses of 3–80 nm. There are four peaks at 2θ = 31.1° and 64.2°, diffracted from the (222) and (444) planes of the ITO film, and at 2θ = 30.4° and 62.8°, diffracted from the (111) and (222) planes of the Y-stabilized ZrO_2 (YSZ) substrate. This highlights that all films used in this work were epitaxial with a (111) out-of-plane orientation.

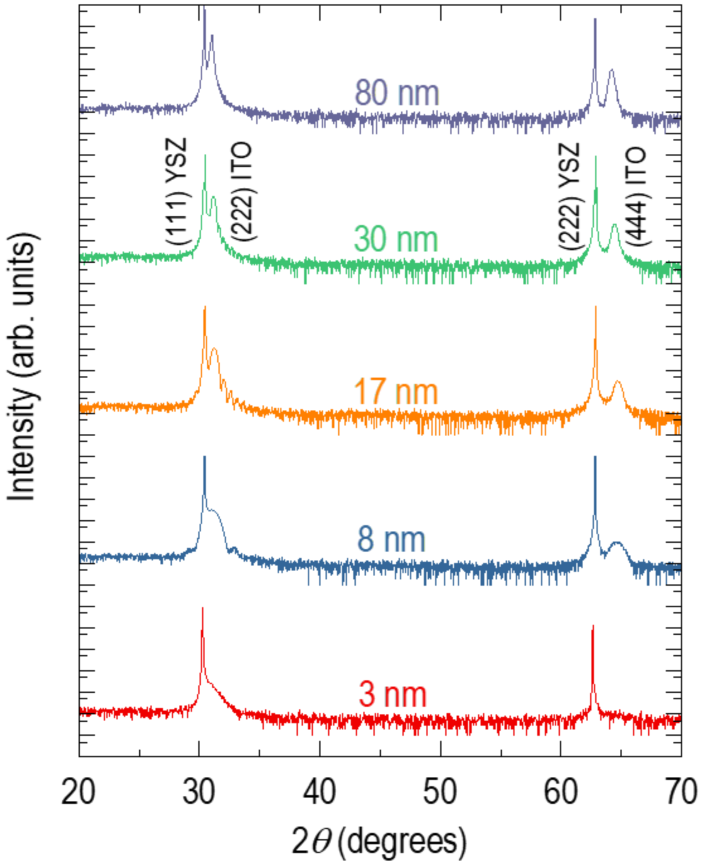

Figure S1. X-ray diffraction θ–2θ scans of various thickness ITO epitaxial films.

2. Existence of defects near ITO/YSZ interfaces

Introducing a minute number of defects can degrade the transmittance and conductivity of transparent electrodes (TEs), as they tend to act as scattering centres. The misfit strain in

epitaxial films has been widely accepted to easily generate more defects near the film/substrate interfaces[1,2]. As mentioned in the paper, our ITO epitaxial films were under in-plane tensile strain with a +1.4% mismatch. Therefore, we investigated the levels of strain and defects with film thickness variation. Figure S2a shows a reciprocal space map around the (113) diffraction peak of YSZ. For the 80-nm-thick film, the bright spot originating from the (226)ITO diffraction has Q_x- and Q_z-values close to the bulk values, indicating that the tensile strain was fully released. With a reduction in thickness, the Q_x-value of the (226)ITO diffraction of the 17-nm-thick film approached the Q_x-value of the (113)YSZ diffraction, indicating that these ITO films were partially under in-plane tensile strain. The 8-nm-thick film showed the same Q_x-values; thus, the ITO films thinner than 8 nm were fully strained. Figure S2b shows X-ray photoemission spectroscopy (XPS) spectra of O-1*s*, Sn-3*d*, and In-3*d* in the ranges of 534–526, 498–484, and 455–441 eV binding energies, respectively[3]. Irrespective of the thickness (8–80 nm), peaks were present at nearly the same binding energy, without a distinguishable change in the spectral shape, indicating homogeneous stoichiometry throughout the thickness. The 3-nm-thick film showed a slight deviation towards a lower binding energy. In this case, a higher density of defects was likely generated near the interface to release the misfit strain, as also evidenced by the transmission electron microscopy (TEM) results (Fig. 1c in the paper).

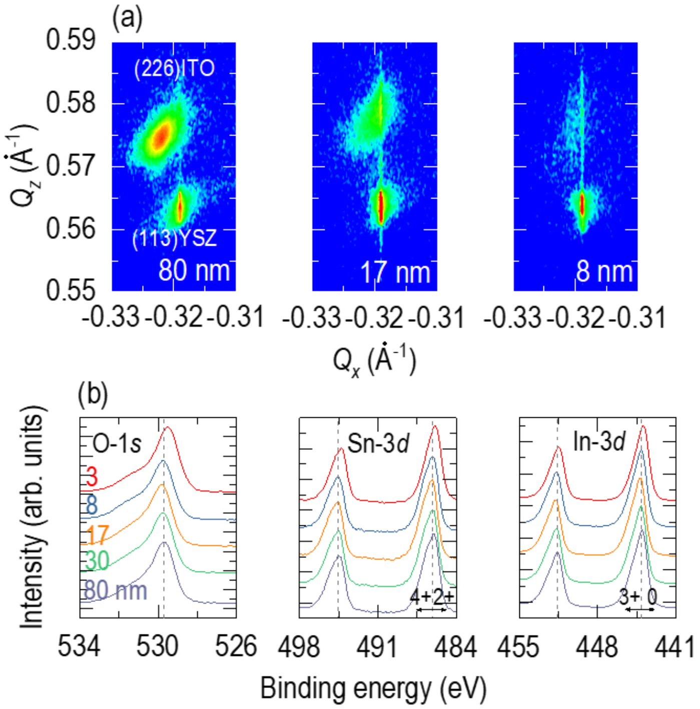

Figure S2. (a) Reciprocal space mapping around (113)YSZ. With a decrease in the film thickness, the Q_x-value of (226)ITO is the same as that of (113)YSZ, indicating fully in-plane tensile-strained films below 8 nm. (b) X-ray photoemission spectroscopy (XPS) spectra of O-1*s*, Sn-3*d*, and In-3*d*. Regardless of the thickness, the peak position and shape are nearly the same over the range of 8–80 nm, indicating that the oxidation states do not significantly change. The 3-nm-thick film shows a peak shift towards lower binding energy, indicating the existence of defects near the interface. The numbers in the XPS spectrum for Sn-3*d* and In-3*d* indicate the oxidation states of Sn and In, respectively[3].

3. Well-ordered atomic arrangement over a wide area and flat film surfaces

We observed a well-ordered atomic arrangement over an ~20-nm-narrow area in the cross-sectional TEM image shown in Fig. 1c. To support this excellent crystallinity, we took cross-sectional TEM images over an ~110-nm-wide area. Figure S3a shows the image for the 80-nm-thick ITO epitaxial film. The atomic arrangement is well ordered, and there are few grain boundaries and dislocations in the region far from the ITO/YSZ interface. It should also be noted that the film surface is very flat, as confirmed by X-ray reflectivity (Fig. 1b). The root-mean-square surface roughness of the 80-nm-thick ITO film is 2.3 nm, as shown in Fig. S3b obtained by atomic force microscopy.

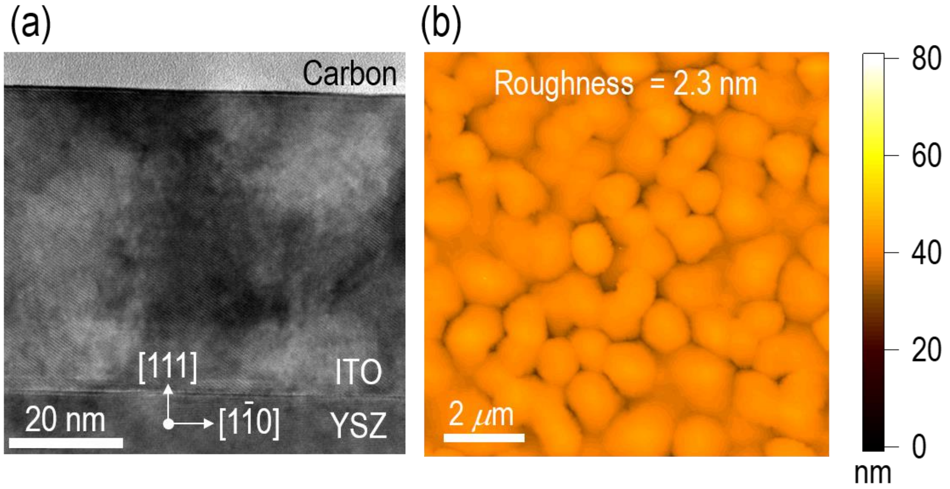

Figure S3. (a) Cross-sectional transmission electron microscopy image and (b) atomic force microscopy surface image of an 80-nm-thick ITO film over a wide area. A smooth surface with a

root-mean-square roughness of 2.3 nm is observed even for the thickest ITO epitaxial films in this work.

4. Metal-to-insulator transition of the 8-, 17-, and 30-nm-thick films

The 8-, 17-, and 30-nm-thick films showed a metal-to-insulator transition (MIT) upon cooling, as shown in Fig. S4. Similar thickness-dependent MIT behaviour has been observed in other materials, including nickelate ultrathin films of 2–3 unit cells[4]. To understand the MIT in ITO epitaxial films, we refer to three scenarios proposed in an earlier study[4]. First, the spatially directional *d* orbitals in Ni^{3+} ions with a $3d^7$ electron configuration are sensitive to strain, which may modify the bond length and oxygen octahedral rotation patterns and amplitudes. However, the overlap of spherically symmetrical In-5*s* orbitals, which are responsible for conduction in ITO, is minimally affected by lattice distortions. Second, quantum confinement is rarely a consideration for our sample, as this MIT behaviour occurs even in 30-nm-thick ITO films. A similar MIT was also observed in 300-nm-thick ITO polycrystalline films[5]. Therefore, another mechanism that is not relevant to strain or quantum effects plays an important role in the MIT of ITO films. Third, defects populate the ITO/YSZ interfaces to release the misfit strain, as evidenced by the TEM (Fig. 1c) and XPS (Fig. S2b) results, and the thermally activated carriers are frozen out at interfacial defects at low temperature. The transition temperature increases with decreasing film thickness (e.g., 100 K for the 30- and 17-nm film thicknesses and 200 K for the 8-nm film thickness), supporting that the MIT of ITO films may be caused by interfacial impurity scattering.

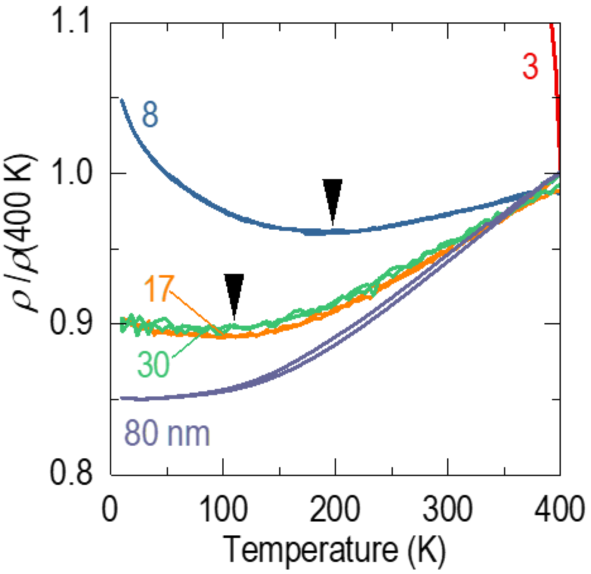

Figure S4. Resistivity as a function of temperature. The resistivity of the 80-nm-thick film decreases with temperature, indicating metallic behaviour. On the other hand, the 8–30-nm-thick films show a metal-to-insulator transition at 100–200 K, as indicated by arrows. The resistivity of the 3-nm-thick film increases as the temperature decreases, indicating insulating behaviour. ρ(400 K) denotes the resistivity at 400 K.

5. Thickness dependence of the transmittance and sheet resistance

We compared the transmittances at 2.5 μm of various thickness ITO, $SrVO_3$, and $CaVO_3$ films, as shown in Fig. S5a. The transmittance of sub-nanometre-thick ITO was 30% higher than those of $SrVO_3$ and $CaVO_3$ and approached 60% for tens-of-nanometre-thick films. The room temperature sheet resistance comparisons, as shown in Fig. S5b, indicated that ITO was sufficiently conductive, but more resistive than $SrVO_3$ and $CaVO_3$.

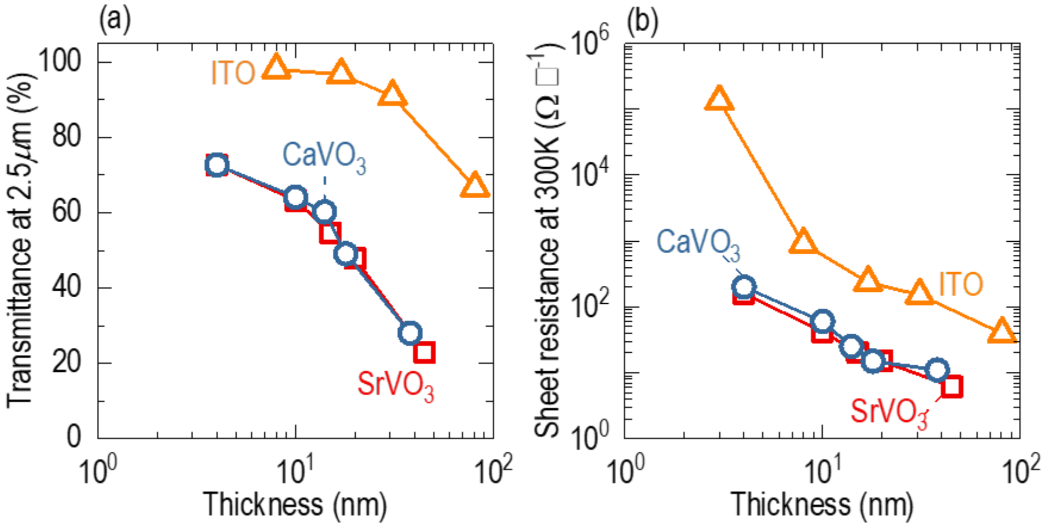

Figure S5. Infrared transparent electrode (IR-TE) performance of ITO epitaxial films. (a) Transmittance as a function of thickness. The transmittance of ITO (measured at the 2.5-μm wavelength) is higher than those of $CaVO_3$ and $SrVO_3$. (b) Sheet resistance as a function of thickness. The sheet resistance (measured at room temperature) of ITO is sufficiently low, except for that of the 3-nm-thick film. We digitized the data of $SrVO_3$ and $CaVO_3$ from reference [6].

6. Calculation of indirect and direct bandgaps

With the assumption of parabolic-like electronic bands, we determined the indirect bandgap and direct bandgap by extrapolation to zero of the linear portion of the sharp rise in the $\alpha^{\frac{1}{2}} \propto (\hbar\omega - E_g)$ and $\alpha^2 \propto (\hbar\omega - E_g)$ curves[7], respectively, where α, $\hbar\omega$, and E_g denote the absorption coefficient, photon energy, and bandgap, respectively (Fig. S6).

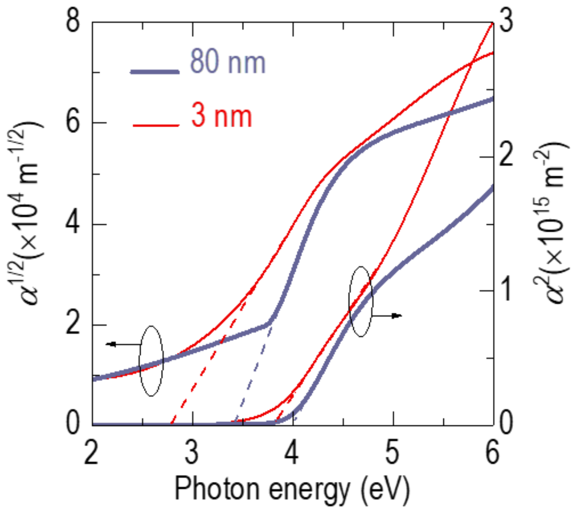

Figure S6. Absorption coefficient as a function of photon energy. By measuring the absorption coefficient, we can calculate the indirect and direct bandgaps by extrapolation to zero of the linear portions of the sharp rises in the absorption coefficient.

References

[1] Aschauer, U., Pfenninger, R., Selbach, S. M., Grande, T. & N. A. Spaldin. Strain-controlled oxygen vacancy formation and ordering in $CaMnO_3$. *Phys. Rev. B* **88**, 054111 (2013).

[2] Mirjolet, M., Vasili, H. B., López-Conesa, L., Estradé, S., Peiró, F., Santiso, J., Sánchez, F., Machado, P., Gargiani, P., Valvidares, M. & Fontcuberta, J. Independent tuning of optical transparency window and electrical properties of epitaxial $SrVO_3$ thin films by substrate mismatch. *Adv. Funct. Mater.* **29,** 1904238 (2019).

[3] Moulder, J. F., Stickle, W. F., Sobol, P. E. & Bomben, K. D. *Handbook of X-ray photoelectron spectroscopy: a reference book of standard spectra for identification and interpretation of XPS data.* (Perkin-Elmer Corporation, Eden Prairie, MN, United States, 1992).

[4] King, P. D. C., Wei, H. I., Nie, Y. F., Uchida, M., Adamo, C., Zhu, S., He, X., Božović, I., Schlom, D. G. & Shen, K. M. Atomic-scale control of competing electronic phases in ultrathin $LaNiO_3$. *Nat. Nanotechnol.* **9**, 443 (2014).

[5] Kaushik, D. K., Kumar, K. U. & Subrahmanyam, A. Metal-insulator transition in tin doped indium oxide (ITO) thin films: quantum correction to the electrical conductivity. *AIP Adv.* **7**, 015109 (2017).

[6] Zhang, L., Zhou, Y., Guo, L., Zhao, W., Barnes, A., Zhang, H.-T., Eaton, C., Zheng, Y., Brahlek, M., Haneef, H. F., Podraza, N. J., Cahn, M. H. W., Gopalan, V., Rabe, K. M. & Engel-Herbert, R. Correlated metals as transparent conductors. *Nat. Mater.* **15**, 204 (2016).

[7] Fox, M. *Optical properties of solids.* (Oxford University Press, Oxford, United Kingdom, 2010).
